# Mortality Rate of COVID-19 With Comorbid Pneumonia in a Rural Area

**DOI:** 10.7759/cureus.63780

**Published:** 2024-07-03

**Authors:** Anmol Multani, Vineesha Kollipara, Tess Krage, James Hearn, Greg Stahl, Kerry Johnson, Scott Goade, Nova Beyersdorfer, Robert D Arnce

**Affiliations:** 1 College of Osteopathic Medicine, Kansas City University, Joplin, USA; 2 Clinical Research, Freeman Health System, Joplin, USA; 3 Mathematics, Missouri Southern State University, Joplin, USA

**Keywords:** mortality, rural, covid-19-induced pneumonia, pneumonia, covid-19

## Abstract

Background: A myriad of risk factors and comorbidities have been determined to influence COVID-19 mortality rates; among these is pneumonia. This study considers pneumonia as a risk factor for increased mortality in patients admitted with COVID-19 in a rural healthcare system. We predicted that the presence of pneumonia of any kind would increase mortality rates in patients admitted with COVID-19.

Methods: A retrospective observational study was conducted using data collected from hospitals in the Freeman Health System (FHS) located in Joplin and Neosho, Missouri. Data were collected between April 1, 2020, and December 31, 2021. Using International Classification of Diseases, Tenth Revision (ICD-10) codes, the investigators identified five distinct patient populations: patients with COVID-19 and pneumonia due to COVID-19 (P1); patients with COVID-19 but without pneumonia due to COVID-19 (P2); patients with COVID-19 and any type of pneumonia (P3); patients with COVID-19 but without any type of pneumonia (P4); and patients without COVID-19 and with any type of pneumonia (P5). In order to understand how pneumonia influences COVID-19 outcomes, the investigators used Wald’s method and a two-sample proportion summary hypothesis test to determine the confidence interval and to compare the mortality rates between these populations, respectively.

Results: The population of patients with COVID-19 and any type of pneumonia (P3) and the population of patients with COVID-19 and pneumonia due to COVID-19 (P1) showed the highest mortality rates. The population of patients with COVID-19 but without any type of pneumonia (P4) had the lowest mortality rate. The data revealed that having pneumonia combined with COVID-19 in any patient population led to a higher mortality rate than COVID-19 alone.

Conclusion: Mortality rates were higher among COVID-19 patients with pneumonia compared to COVID-19 patients without pneumonia. Additionally, pneumonia, by itself, was found to have a higher mortality rate compared to COVID-19 alone.

## Introduction

According to the Centers for Disease Control and Prevention, over one million people in the United States have died due to COVID-19 since January 21, 2020 [1]. This pandemic demonstrated the risk of young individuals contracting the virus. A Houston, Texas, study investigating risk factors for severe disease among young adults (ages 18-29 years) diagnosed in the hospital with COVID-19 revealed that 17% of patients were diagnosed with pneumonia within 30 days of their initial hospital admission [2]. Additionally, the study identified associations between conditions such as asthma, diabetes, and obesity and an increased likelihood of pneumonia occurrence [2]. Non-Hispanic Asian or Hispanic race/ethnicity was also found to be a factor associated with higher odds of having pneumonia [2].

According to an analysis by the National Rural Health Association based on county-level data as of October 23, 2020, mortality rates due to COVID-19 differ depending on the location of the patient [3]. The analysis reported COVID-19 mortality rates of 65.43 per 100,000 for urban counties and 50.78 per 100,000 for rural counties [3]. However, more focused studies of COVID-19 mortality rates in rural and urban settings may reflect higher mortality risks among rural populations. In a retrospective cohort analysis of one North Carolina hospital, it was found that rural patients experienced a higher rate of death or discharge to hospice, with 16.5% of rural patients affected compared to 13.3% in the urban cohort [4]. This analysis demonstrated how regional policy, circumstances, and resources subject rural populations to greater COVID-19 mortality risks. These risks included higher rates of comorbidities, lower life expectancies, increased age, less access to healthcare, and inadequate medical technology or resources in rural settings [4,5].

All the foregoing factors contribute to differential outcomes among COVID-19 patients, and these outcomes include the development of severe diseases, such as pneumonia. In a study comparing rates of bacterial infections and mortality in COVID-19 patients with pulmonary infiltrates alongside patients who were diagnosed with pneumonia the year prior, it was determined that mortality rates remained elevated in COVID-19 patients compared to non-COVID-19 individuals, even following adjustments for factors such as age, tachypnea, hypoxemia, and bacterial infection [6].

This difference in mortality can further be applied to populations with other risk factors, and our study analyzed mortality rates in a rural population. Determining risk factors for mortality rates in rural COVID-19 hospitalized patients is vital to implementing policy and targeting comorbid dangers unique to underserved populations. For example, a free online health tool, the COVID-19 ED pneumonia mortality index, or CoV-ED-PMI, was created and verified through the analysis of clinical information from COVID-19 patients admitted to the emergency department, enabling the prediction of mortality within a month [7]. These data are crucial in developing methods and measures to reinforce the work of rural intensive care units and to provide resources to these healthcare systems. Based on the demonstrated association between COVID-19-induced pneumonia or comorbid severe disease and mortality rates, along with risk factors for rural patient populations, the investigators predict increased mortality rates among patients with both comorbid pneumonia and COVID-19-induced pneumonia.

## Materials and methods

Data source

Patient data were collected from patients aged 18 years or older who were admitted to Freeman Health System (FHS) in Joplin and Neosho, Missouri, between April 1, 2020, and December 31, 2021. Data were extracted from the electronic medical records of patients. The protocol was approved by the Institutional Review Board of FHS under the title: Risk Factors for Poor Outcomes in Patients Admitted with the Diagnosis of COVID-19 Infection #2022001. Since the study had a retrospective design, informed consent was not required.

Data selection and defined groups

**Figure 1:**
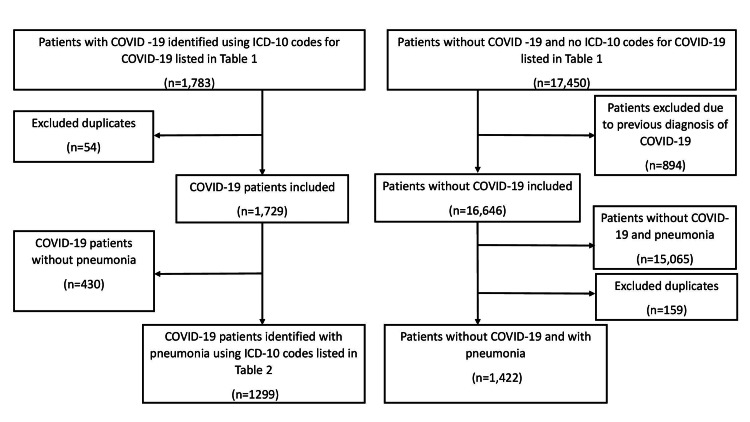
Flowchart showing selection of patients from hospital database into defined groups delineating COVID-19 status and the presence of pneumonia

Patients with COVID-19 were identified using the International Classification of Diseases, Tenth Revision (ICD-10) diagnosis code listed in Table 1. As shown in Figure 1, a total of 1,783 patients with COVID-19 were identified, and 1,729 patients were included after removing 54 duplicate admissions. From this initial sample of COVID-19 patients, 1,299 patients were included in the pneumonia population using the ICD-10 codes listed in Table 2, and 430 patients who did not have pneumonia ICD-10 codes were eliminated. The investigators identified patients who did not have the ICD-10 code for COVID-19 (U07.1). A total of 17,540 patients were identified without COVID-19. There were 894 patients who had previous COVID-19 admissions and were excluded from the initial pool. A total of 16,646 patients were included in the population of patients without COVID-19. Of the 16,646 cases without COVID-19, 1,422 patients had pneumonia without COVID-19. Table 2 lists their ICD-10 codes. Excluded were 159 duplicate cases and 15,065 cases without pneumonia and COVID-19. Wald’s method was employed to determine sample proportions for mortality rates of COVID-19 with pneumonia as a comorbidity, pneumonia due to COVID-19, and pneumonia without COVID-19. Patient data were segregated into five disease categories (P1 to P5) using ICD-10 codes, as described in Table 3. We used StatCrunch software by Pearson Education Inc., Hoboken, NJ, USA, to conduct a two-sample proportion test to compare mortality rates, as a primary outcome, between COVID-19 patients with any type of pneumonia (pneumonia due to COVID-19 or as a comorbidity) and COVID-19 patients without pneumonia. In the two-sample proportion test, raw data points were expressed as “Y” or “N,” indicating whether the patient died or not. The null hypothesis was that the two groups being compared had no difference in proportions of mortality. The two-sample proportion test generated a test statistic, and using the normal (z) distribution, P-values were found. P < 0.05 was considered significant.

**Table 1:** ICD-10 code for COVID-19

COVID-19 ICD-10 Codes	Diagnosis
U071	COVID-19

**Table 2:** ICD-10 codes for pneumonia

Pneumonia ICD-10 Codes	Diagnosis
B961	*Klebsiella pneumoniae* as the cause of diseases classified elsewhere
J1008	Influenza due to other identified influenza virus with other specified pneumonia
J1108	Influenza due to unidentified influenza virus with specified pneumonia
J1281	Pneumonia due to SARS-associated coronavirus
J1282	Pneumonia due to coronavirus disease 2019
J1289	Other viral pneumonia
J129	Viral pneumonia, unspecified
J13	Pneumonia due to *Streptococcus pneumoniae*
J14	Pneumonia due to *Hemophilus influenzae*
J150	Pneumonia due to *K. pneumoniae*
J151	Pneumonia due to Pseudomonas
J1520	Pneumonia due to Staphylococcus, unspecified
J15211	Pneumonia due to Methicillin-susceptible *Staphylococcus aureus*
J15212	Pneumonia due to Methicillin-resistant *Staphylococcus aureus*
J153	Pneumonia due to streptococcus, group B
J154	Pneumonia due to other streptococci
J155	Pneumonia due to *Escherichia coli*
J156	Pneumonia due to other Gram-negative bacteria
J157	Pneumonia due to *Mycoplasma pneumoniae*
J158	Pneumonia due to other specified bacteria
J159	Unspecified bacterial pneumonia
J168	Pneumonia due to other specified infectious organisms
J17	Pneumonia in diseases classified elsewhere
J188	Other pneumonia, unspecified organism
J189	Pneumonia, unspecified organism
J851	Abscess of lung with pneumonia
J95851	Ventilator associated pneumonia

**Table 3:** Patient groups based on the presence and absence of pneumonia and COVID-19

	Populations	ICD-10 codes used
P1	Patients with COVID-19 and pneumonia d/t COVID-19	U071 and J1282
P2	Patients with COVID-19 and without pneumonia d/t COVID-19	U071 without code J1282
P3	Patients with COVID-19 and any type of pneumonia	U071 and all the pneumonia codes listed in Table 2
P4	Patients with COVID-19 and without any type of pneumonia	U071 without any of the pneumonia codes listed in Table 2
P5	Patients without COVID-19 and with any type of pneumonia	ICD-10 codes listed in Table 2 without code U071

## Results

**Figure 2:**
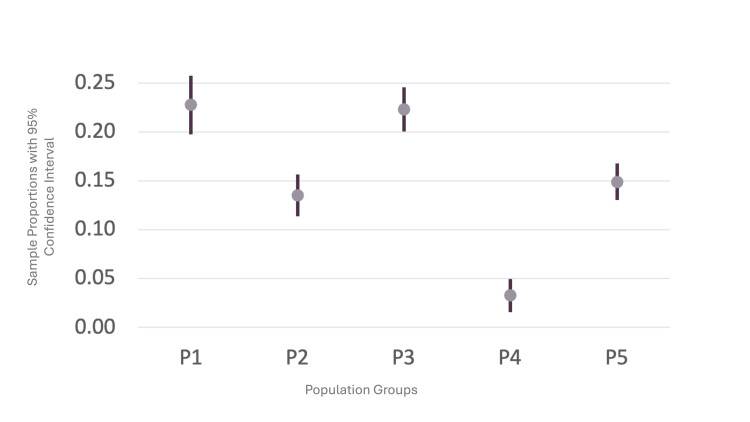
Mortality rates of population groups with confidence intervals

**Figure 3:**
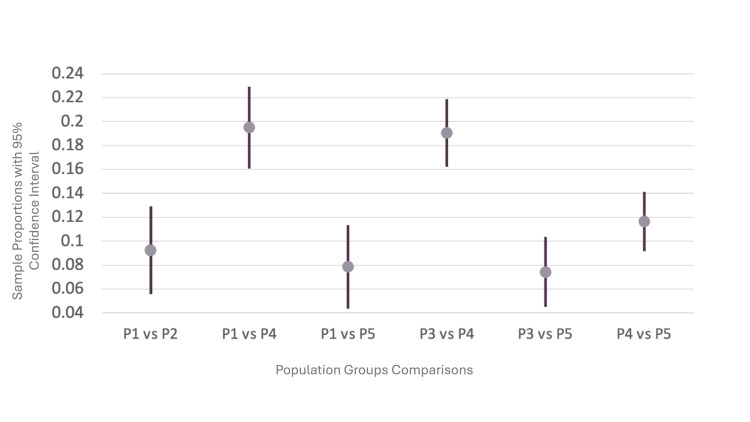
Comparisons of mortality rates of different population groups with confidence intervals

Using two-sample proportion tests, the mortality of all five populations was compared. The results demonstrated that the two groups with the highest mortality rates were P3 (patients with COVID-19 and any type of pneumonia) and P1 (patients with COVID-19 and pneumonia due to COVID-19), as noted in Table 4. These results are reflected in Figure 2, along with the similarities in mortality rates among the five populations. The group with the lowest mortality rate was P4 (patients with COVID-19 and without any type of pneumonia). Figure 3 provides the comparisons noted in Table 5 and shows the confidence intervals (CI) in the difference in mortality rates. The data reveal that any combination involving pneumonia (P1, P3, and P5) leads to a higher mortality rate than COVID-19 without any type of pneumonia (P4).

**Table 4:** Mortality rates of each patient group

Populations	Mortality	Sample proportion	Lower 95% CI	Upper 95% CI
P1	173 of 760	0.2276	0.1978	0.2574
P2	131 of 969	0.1352	0.1137	0.1567
P3	290 of 1299	0.2232	0.2006	0.2459
P4	14 of 430	0.0326	0.0158	0.0493
P5	212 of 1422	0.1491	0.1306	0.1676

**Table 5:** Comparisons of mortality rates of different patient groups

Comparisons	Sample 1	Sample 2	P1-P2	Lower 95% CI for P1-P2	Upper 95% CI for P1-P2	P-value
P1 vs. P2	Mortality	Mortality	0.0924	0.0557	0.1292	<0.001
	173 of 760	131 of 969				
	(0.2276)	(0.1352)				
P1 vs. P4	Mortality	Mortality	0.1951	0.1609	0.2293	<0.001
	173 of 760	14 of 430				
	(0.2276)	(0.0326)				
P1 vs. P5	Mortality	Mortality	0.0785	0.0435	0.1136	<0.001
	173 of 760	212 of 1422				
	(0.2276)	(0.1491)				
P3 vs. P4	Mortality	Mortality	0.1907	0.1625	0.2189	<0.001
	290 of 1299	14 of 430				
	(0.2232)	(0.0326)				
P3 vs. P5	Mortality	Mortality	0.0742	0.0449	0.1034	<0.001
	290 of 1299	212 of 1422				
	(0.2232)	(0.1491)				
P5 vs. P4	Mortality	Mortality	0.1165	0.0915	0.1415	<0.001
	212 of 1422	14 of 430				
	(0.1491)	(0.0326)				

## Discussion

The objective of this study was to identify whether patients with both pneumonia and COVID-19 have higher mortality rates than those without the comorbidity of pneumonia, focusing on a rural population. The initial hypothesis was that having pneumonia as a comorbidity or as a complication of COVID-19 would negatively influence the prognosis for COVID-19 patients and lead to increased mortality. The results of this study supported the hypothesis. The statistical analysis of the collected data demonstrates that any combination of COVID-19 and pneumonia leads to a higher mortality rate than COVID-19 or pneumonia alone.

The data revealed that there was a statistically significant increase in mortality in patients with COVID-19 and pneumonia due to COVID-19 compared to patients with COVID-19 without pneumonia due to COVID-19 (P2), patients with COVID-19 without any pneumonia (P4), and patients with pneumonia without COVID-19 (P5). These results further suggest that pneumonia is a greater contributor to death among the two conditions, as seen by the P4 population (patients with COVID-19 and without any type of pneumonia), which has the lowest mortality rate between 1.58% and 4.93%. These results have important implications for the formulation of policies relating to the allocation of resources to pneumonia due to its lethality. Additionally, it may be advisable to revise protocols for the treatment of patients presenting with pneumonia and COVID-19.

The results of the study were consistent with findings from previous research. Patients infected with COVID-19 who also develop pneumonia are more likely to require non-invasive ventilation and oxygen supplementation during their hospitalization and develop complications more frequently [8,9]. In fact, the presence of severe pneumonia is an independent factor contributing to higher mortality in COVID-19 patients [10]. One of the studies found that COVID-19 pneumonia patients had a 6.6% mortality rate and longer hospital stays compared to COVID-19 patients without pneumonia who did not have any mortality [9]. Like our results, previous studies support the idea that COVID-19 pneumonia has a worse prognosis than pneumonia from other infections, including bacterial, fungal, viral, and atypical infections [11]. One of the reasons for that is the longer clinical course of COVID-19 pneumonia, which ranges from six to twelve days. Pneumonia due to influenza, on the other hand, lasts up to seven days. Inflammatory cytokines, like IL-6, rise during the duration of the illness, and prolonged exposure to them may lead to a greater extent of immunopathology in COVID-19 pneumonia patients [12]. Elevated cytokines activate endothelial cells and trigger progressive thrombosis, microvascular dysfunction, and widespread coagulopathy, which contribute to the pathogenesis of severe pneumonia [12]. Alveolar integrity is also impacted diffusely in COVID-19 pneumonia due to SARS-CoV-2’s affinity for type II epithelial cells, which are responsible for surfactant production and prevent the collapse of alveoli using surfactant [11]. Our results indicate that any combination of COVID-19 and pneumonia leads to a worse prognosis. One scenario is pneumonia due to COVID-19. Other scenarios include patients developing secondary bacterial or viral superinfections of the lungs after COVID-19 [11]. In previous studies, 12% of COVID-19 patients had confections, and 14% had superinfections [10]. Infection with SARS-CoV-2 can damage lymphocytes and impair the immune system, increasing susceptibility to bacterial and other viral infections. This can play a role in increasing disease severity and requiring intensive care measures [11,13]. With suppression of immunity and viral replication in the lower respiratory tract, greater lung injury results in a higher risk of mortality associated with COVID-19 [11,14].

This study has certain limitations, which may have impacted the results. It did not consider the presence of comorbidities other than COVID-19, which may have impacted the outcome of pneumonia in patients, including asthma, diabetes, and obesity. It is possible that the patients in the different subgroups of pneumonia and COVID-19 had similar comorbidities, which could, in turn, influence their vulnerability, disease progression, and mortality. Future investigators should perhaps further stratify patients based on other comorbidities and analyze how they might be interacting with COVID-19 and pneumonia. Since the results of this study were based on patient data collected from two midwestern hospitals within one health system in a rural setting, it is uncertain whether the results can be applied to patients throughout the United States, and it appears that combining patient data collected from both urban and rural hospitals throughout the country would produce results of greater value. This study also did not consider the differences in socioeconomic factors between study subjects, which have previously been shown to affect the COVID-19 prognosis. Other social factors, such as geographical location, access to care, and education, may also contribute to an increase in adverse COVID-19 outcomes. These factors may function as confounding variables. Thus, future research should take them into account to determine if they may be influencing COVID-19 progression and the severity of the illness. Further, the patient data from the hospitals were not collected for purposes of COVID-19 research, and therefore, it reported all-cause mortality rather than mortality specific to COVID-19 or pneumonia. This may have resulted in misclassification bias, in which errors can be made in assigning diagnoses to patients based on their clinical presentation and other pulmonary testing. This can alter the overall population size and mortality rate of each of the subgroups. Given the retrospective nature of the study, conclusions can be drawn regarding the association of pneumonia and COVID-19 with higher mortality; however, causation cannot be established. Finally, the sample was not chosen at random, and it is unable to be determined if the sample analyzed is representative of the population. Collectively, these conditions explain the inability to analyze additional factors among subjects.

Future research should be conducted to determine how to modify COVID-19 protocols in patients with comorbid pneumonia to focus primarily on treating pneumonia to decrease mortality rates. The primary objective of this study was to determine the impact of pneumonia on COVID-19 mortality in a rural population, which was accomplished. These results can be used to further tailor patient care, reduce mortality due to comorbid pneumonia in COVID-19 patients, and improve overall patient outcomes. Future studies may explore how hypoxemia in pneumonia patients influences the COVID-19 prognosis. The degree of hypoxemia has been shown to be an independent predictor of COVID-19 mortality [15]. It is also possible to study how pneumonia affects oxygen saturation in COVID-19 patients to reduce survival and identify patients with hypoxemia and pneumonia to provide early access to care and reduce COVID-19-related deaths. Another proposed method of distinguishing COVID-19 from other infective types of pneumonia is hypocalcemia [16]. Likewise, glycemic control in COVID-19-induced pneumonia patients was examined in comparison to their non-COVID-19 pneumonia counterparts to assess the outcomes of critical illness based on hyperglycemia [17]. Any measure that exists to stratify the risk of severe disease among COVID-19 patients can reduce mortality rates among hospitalized COVID-19 patients.

## Conclusions

In conclusion, this study highlights the intricate interplay between COVID-19 and pneumonia, emphasizing the heightened risk of mortality associated with their co-occurrence, especially within rural populations. There is a statistically significant increase in mortality between patients with COVID-19 with any pneumonia (P3) and those with COVID-19 but without any pneumonia (P4). Additionally, the results reflect that any combination involving pneumonia leads to a higher patient mortality rate than COVID-19 itself, implying that pneumonia may be a deadlier condition than COVID-19 in this rural population. The findings underscore the need for tailored healthcare policies and resource allocation strategies to address the distinct challenges faced by rural communities in managing COVID-19. Furthermore, the study identifies crucial avenues for future research, including the refinement of treatment protocols to prioritize pneumonia management in COVID-19 patients. By leveraging these insights, healthcare providers can enhance patient care protocols, optimize resource utilization, and ultimately mitigate the disproportionate impact of COVID-19 on vulnerable populations.
